# Efficacy and mechanism of retinyl palmitate against UVB-induced skin photoaging

**DOI:** 10.3389/fphar.2023.1278838

**Published:** 2023-10-20

**Authors:** Peng Shu, Menggeng Li, Nan Zhao, Yuan Wang, Lanyue Zhang, Zhiyun Du

**Affiliations:** ^1^ State Key Laboratory Basis of Xinjiang Indigenous Medicinal Plants Resource Utilization, CAS Key Laboratory of Chemistry of Plant Resources in Arid Regions, Xinjiang Technical Institute of Physics and Chemistry, Chinese Academy of Sciences, Urumqi, Xing Jiang, China; ^2^ HBN Research Institute and Biological Laboratory, Shenzhen Hujia Technology Co., Ltd., Shenzhen, Guangdong, China; ^3^ University of Chinese Academy of Sciences, Beijing, China; ^4^ School of Biomedical and Pharmaceutical Sciences, Guangdong University of Technology, Guangzhou, Guangdong, China

**Keywords:** retinyl palmitate, UVB induced photoaging, skin healing, anti-aging, vitamin A derivative

## Abstract

Retinyl palmitate (RP) is a vitamin A derivative that has been widely used in anti-aging and skin treatment. The aim of this study is to investigate the effect of RP on UVB (Ultraviolet radiation B) induced photoaging and its potential mechanism. Immunofluorescence assay demonstrates that RP can reduce collagen degradation in skin cells by UVB radiation and reduce apoptosis of skin cells. Cell migration assay reveals that RP can increase cell migration rate, helping to repair skin damage and restore cell viability. Immunohistochemical assays indicate that RP can significantly reduce the expression of IL-6, IL-1β, TNF-α induced by UVB radiation. Moreover, metabolomics and transcriptomics results suggest that RP regulates several metabolic pathways and gene expression, particularly in inflammatory signaling pathways, collagen synthesis and apoptosis, exhibiting significant regulatory effects. Furthermore, network pharmacological analysis predicts that RP may affect UVB-induced photoaging by regulating multiple key proteins and signaling pathways. Overall, this study demonstrates that RP has significant anti-photoaging ability, acting through several pathways including inhibition of inflammatory response, promotion of collagen synthesis and inhibition of apoptosis. These results provide a scientific basis for the application of RP in skin anti-photoaging and therapy, enabling the potential usage of RP to skin care products.

## 1 Introduction

Skin aging is a complex process influenced by several endogenous and exogenous factors (Miean and Mohammed, 2001; Kang et al., 2010; Wang et al., 2012). Photoaging is an important aspect of the skin aging process, and UVB exposure is one of the main exogenous factors contributing to photoaging (Fisher et al., 1997; Rittié and Fisher, 2015; Gromkowska-Kępka et al., 2021). UVB radiation is able to directly penetrate the atmosphere and interact with cells in the skin. Oxidative stress is considered to be one of the main mechanisms of UVB-induced photoaging of the skin (Ikehata and Ono, 2011; Chen et al., 2021). UVB radiation generates large amounts of free radicals and reactive oxygen molecules, causing intracellular oxidative stress, which in turn damages important biomolecules such as cell membranes, fibronectin and DNA, leading to a decrease in cellular function and thinning of the epidermal layer (Pinnell, 2003; Kammeyer and Luiten, 2015; Chen et al., 2021). In addition, UVB irradiation activates the release of various inflammatory cells and inflammatory factors, such as tumor necrosis factor-alpha (TNF-α) and interleukin-1 (IL-1), accelerating the skin aging process. Long-term exposure to UVB radiation can cause adverse changes such as uneven skin texture, wrinkle formation, decreased elasticity and skin pigmentation, which can have serious effects on the health and appearance of the skin (Bennett et al., 2008; Bucay et al., 2020; Gromkowska-Kępka et al., 2021). Therefore, it is of great theoretical and practical importance to study the role of UVB induced skin photoaging and its mechanisms.

In order to understand and prevent UVB induced photoaging of the skin, many studies have focused on finding effective anti-photoaging strategies (Han et al., 2014; Guan et al., 2021; Krutmann et al., 2021). These strategies include the use of chemicals (Lee, 2016; Bacqueville et al., 2022), natural substances (Tran et al., 2021; Wang et al., 2022), and photoprotectants (Fardiyah et al., 2020; Kasanah et al., 2022) to reduce skin damage from UVB radiation and inhibit the onset of photoaging. In addition, several cellular studies and clinical studies are underway to find new molecular targets and therapeutic approaches for the treatment and reversal of UVB induced skin photoaging (Gardner and Weiss, 1990; Ho and Dreesen, 2021; Tang et al., 2021; Battie and Verschoore, 2022; Callender et al., 2022).

Vitamin A is an essential and vital substance for life activities and is an essential class of micronutrients that plays an important role in maintaining visual health, epithelial cell integrity, involvement in growth and reproduction, anti-tumor and maintaining the integrity of the immune system (Baldwin et al., 2013; Huang et al., 2018; Polcz and Barbul, 2019). Retinoic acid, the active form of vitamin A, has been extensively studied and used in skin care. Several early studies have shown that retinoic acid has anti-aging activity on the skin; for example, in addition to its antioxidant activity, retinoic acid induces epidermal thickening, inhibits UV induced matrix metalloproteinases, and promotes collagen synthesis in photoaged skin (premature skin aging induced by ultraviolet light, 1997). However, retinoic acid is irritating to the skin and prone to adverse reactions after application. Compared to retinoic acid, retinol and retinyl esters are more widely used in cosmetics, and their mechanism of action is that retinyl esters penetrate into the skin and are transformed into retinol by the action of esterases, then into retinal by the action of enzymes, and finally into retinoic acid. This process prevents strong skin irritation and activates cell renewal. Due to its chemical nature, retinol tends to decompose when exposed to UV light, high temperatures or the presence of oxygen (Lian et al., 2017). Therefore, retinyl palmitate, a more stable esterified form of retinol, is considered as an alternative to retinol and is used as an active ingredient in pharmaceuticals and cosmetics as an effective anti-aging agent (Ro et al., 2013).

Retinol palmitate is A derivative of vitamin A, also known as vitamin A palmitate, which is easily absorbed by the skin and converted into retinol. The main function of retinol palmitate is to accelerate skin metabolism, promote cell proliferation, and stimulate collagen production (Senoo et al., 1996), which also has a certain effect on the treatment of acne (Algahtani et al., 2020). At present, because it has been proven to have a strong antioxidant effect of clearing free radicals (Xia et al., 2006), this ingredient was used as the preferred ingredient of antioxidant and anti-aging by skin care products or cosmetics, allowing the addition of no more than 1% in skin care products.

Although some studies have suggested that retinyl palmitate may exert its anti-photoaging effects by inhibiting inflammatory responses, reducing oxidative stress and regulating gene expression (National Toxicology Program, 2012; Rawlings et al., 2013; Chien et al., 2022). However, the specific mechanism of action of retinyl palmitate on UVB induced photoaging is not fully understood. Therefore, this study was conducted to understand the effects of UVB irradiation on skin epidermal and dermal structure and metabolism by establishing an *in vitro* photoaging model and an animal model of photoaging. Including the effects of retinyl palmitate on epidermal cell structure and proliferation, the level of oxidative damage and protein expression, the aim was to investigate in depth the protective and action mechanisms of retinyl palmitate on UVB induced structural and various metabolic alterations in the epidermis and dermis in order to better understand its anti-photoaging effects. By studying the action mechanism of retinoic acid palmitate, we can provide new ideas and new directions for the development of innovative treatments and skin care products for photoaging, which is conducive to the study of anti-photoaging strategies and help people to make effective preventive measures. Adopt scientific methods to alleviate skin problems caused by UV-induced photoaging.

## 2 Materials and methods

### 2.1 Materials

Retinyl palmitate was purchased from Dutch State Mines (DSM). MTT (thiazolium blue), DMEM (dulbecco’s modified eagle medium), MEM (minimum essential medium), PBS (phosphate buffered saline) buffer, trypsin, double antibody, β-galactosidase cell aging kit were purchased from Aladdin.

### 2.2 Instruments

UVB lamp was used for UVB induced photoaging mouse models. Nikon Eclipse TE200 fluorescence microscope (Nikon Company, Japan) and Multiskan FC enzyme labeling instrument (Semefeld Company, United States) were used for analysis.

### 2.3 Cell viability was measured by MTT assay

HaCat cells were seeded in 96-well plates at a 24 h density, cells adhered to the wall and treated with different concentrations of RP (0.625, 1.25, 2.5, 5, and 10 μg/mL), respectively, and cells were incubated for 24 h. The blank group and the control group were added with the same amount of thiobarbital sodium and culture medium respectively, with 5 repeats in each group. 24 h later, the prepared tetramethylazolium salt solution was diluted to 0.5 mg/mL with a medium, 100 μL was added to each well, wrapped in tin foil and incubated in a dark incubator. After 4 h, the diluent of tetrazolium salt or sodium thiobarbital was discarded with the tissue and 100 μL low density lipoprotein was added. After concussion, RP absorption (570 Nm) was measured by enzyme labeling, and cell survival was calculated (Zhou et al., 2012; Kumar et al., 2018).

### 2.4 Immunofluorescence

The concentration of HaCat cells was adjusted to 1 × 105 cells/mL, and 1 mL cell suspension was added to each well of 24-well plate. After 24 h of culture, the supernatant was discarded, 1 mL of PBS was added, and the model was made with UVB (the modeling dose was 300 mJ/cm2). Different concentrations of 1 mL medium were added to each well and placed in the 5% CO2 incubator for cell culture. After 24 h, the supernatant was discarded and washed with PBS. Add 4% paraformaldehyde to fix 15 min, add 0.5% TritonX-100 to permeable 10min, and seal with 5% BSA at room temperature for 1 h. The 24-well plate was incubated with PPAR (peroxisome roliferator activated receptor) antibody (1 pur 200) 200 μL per well overnight at 4, then washed with TBST (Total Billing System + Tween), and the antibody was prepared with 1% BSA. The 24-well plate was incubated at room temperature and hidden from light with a second fluorescent antibody for 1 h, and then washed with TBST. 10 min was incubated with DAPI dye solution (200 μL) and detected by inverted fluorescence microscope (Mu et al., 2021).

### 2.5 Cell migration

For the experiment of cell migration, after cell passage, the cell count was carried out, and the concentration of cell suspension was adjusted to 2 × 105 cell/mL. Cells were inoculated in 48 well plates and 1 mL was inoculated in each well. The culture plate was put into 5% CO2 incubator at 37 for cell culture. After 24 h, the supernatant was discarded and the cells were washed. The basic medium containing different concentrations of drugs was added, and a blank control group was set up, with 3 compound holes in each group. Concentration gradient settings: 2.5, 5, and 10 μg/mL. The culture plate was put into 5% CO2 incubator at 37 for cell culture. At 0 when RP was added, cell-scattering images were taken under microscope at 0, 6, 12, and 24 h, and four visual fields were selected for each well. Results were obtained from three randomly selected high power visual fields, and mean values were determined from three repeated experiments. Relative mobility = (initial scratch width-scratch width after culture)/initial scratch width × 100% (Wu et al., 2012).

### 2.6 Animals

25 KM SPF grade mice, male, 5 weeks old, weighing 34–38 g, were purchased from Guangdong Experimental Animal Center. These mice were adapted for 4 days in a 12 h light/dark cycle at 22°C. 25 Mice were randomly divided into blank group (control group), model group (model group), 0.025% RP (C-RP-L), 0.05% RP (C-RP-M) and 0.1% RP (C-RP-H). On the day before the experiment, the back of mice was treated with hair removal cream, and the depilation area was about 4 cm × 4 cm. Except for the control group, all mice were exposed to UVB (100 mJ/cm2) once daily for 1 week. After the model was established, neither the model group nor the control group did any treatment. The mice in the experimental group were smeared 0.025%, 0.05%, 0.1% RP 200 μL on the back of the hairless part, respectively, once a day. The back skin tissue was fixed with 4% paraformaldehyde for HE staining, TB staining, Masson’s trichromatic staining, and immunohistochemical staining (Wang et al., 2019).

### 2.7 Hematoxylin and eosin (HE) staining

The proliferation of skin epidermis was observed by hematoxylin-eosin staining. The skin tissue on the back of the mice was secured with a 10% tissue fastener and embedded in paraffin. Paraffin sections were dewaxed with xylene (10 min), anhydrous ethanol (10 min) and 75% ethanol (5 min) to water. It is dyed, dried and sealed with transparent and neutral glue. Histological changes were observed under a light microscope, images were collected, and epidermal thickness was measured by IMAGE Pro PLUS software (Cui et al., 2022).

### 2.8 Toluidine blue staining

The number of mast cells in skin tissue was counted by toluidine blue staining. The paraffin sections were soaked in xylene (40 min), anhydrous ethanol (30 min) and 75% ethanol (5 min) for dewaxing into water. Toluidine blue staining: animal tissue sections were soaked in dye solution (2–5 min), washed with clean water, differentiated by 0.1% glacial acetic acid, terminated by running water washing, and the degree of differentiation was controlled under microscope. After washing with tap water, put the slices in the oven to dry. Transparent seal: The slice is placed in a clean transparent xylene box and 10 min is sealed with neutral film. Microscope, image acquisition and analysis.

### 2.9 Masson staining

Paraffin sections were dewaxed with water, fixed with Bouin’s solution, stained with sky blue for 2–3 min, washed, stained with hematoxylin staining solution for 2–3 min, differentiated with 1% hydrochloric acid ethanol for a few seconds, soaked in rinse for 10 min, and then stained with fuchsin solution. Microscope, image acquisition and analysis (He et al., 2020).

### 2.10 Immunohistochemical staining

The paraffin sections of mouse skin were stained with immunohistochemistry. The paraffin sections were dewaxed with xylene (10 min), anhydrous ethanol (10 min) and 75% ethanol (5 min) to water and soaked in citric acid antigen repair solution for antigen repair. After washing, 3% hydrogen peroxide was added to block endogenous peroxidase. The tissue ring was evenly covered with 3% BSA to block the serum. After sealing, the corresponding first and second antibodies were added and incubated, and finally color development and reverse hematoxylin staining were performed. Finally, the slices were gradually dehydrated in 75% alcohol, 85% alcohol and anhydrous ethanol, and then placed in xylene for 5 min. After drying, seal with transparent neutral glue. Image-ProPlus was used to measure the data, and GraphpadPrism was used to analyze and plot the data. The indexes measured in the experiment were IL-6, IL-1β, TNF-α and Col-I. The measure index is based on normal index of blank group and outlier index of UVB model group. After medication, observe the change trend of each index in this interval, and judge the actual influence trend of a certain index.

### 2.11 Metabolomics, transcriptomics and network pharmacology

#### 2.11.1 Retinol palmitate gene card database targets

Ten targets of RP were predicted by Swiss Target Prediction (Swiss Target Prediction < http://www.swisstargetprediction.ch/index.php >). With “skin aging” as the key word, the Gene Cards database was searched, and the targets of skin aging were obtained. There were 19578 results after weight loss. The results were then uploaded to the online VENN map and submitted, and a total of 10 intersection targets were obtained.

#### 2.11.2 Metabolite, transcriptomics and gene set enrichment, pathway and molecular network analysis

For each gene, its basic function is based on its protein domain and the literature studied. GO and KEGG are databases of gene-related functions stored based on different classification ideas. With the help of DAVID website [DAVID: Functional Annotation Tools (ncifcrf.gov)], GO enrichment analysis and KEGG analysis were used to screen the signal pathway. After uploading the common protein gene to STRING website (www.string-db.org/), a preliminary information map of protein interactions was obtained. In addition, the compound-action target network is constructed using Cytoscape 3.7.2 software to screen core nodes according to network topology characteristics such as node value. Metabolomics, transcriptomics and network pharmacology (22).

### 2.12 Statistical analysis

The results are expressed in the form of mean ± standard deviation (SD) of triple values. The statistical difference between averages was determined by Tukey’s one-way ANOVA test using GraphpadPrism8 statistical software. *p* < 0.05, the difference was statistically significant.

## 3 Result and discussion

### 3.1 Cell experimental analysis

#### 3.1.1 MTT cytotoxicity assay analysis

**FIGURE 1:**
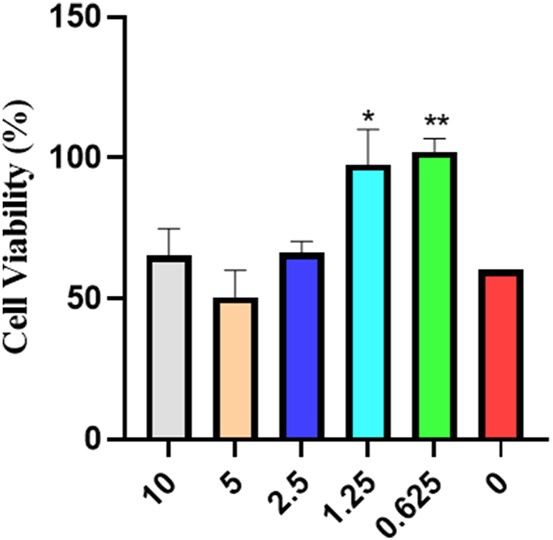
MTT cytotoxicity assay analysis of cell viability (Compared to the model group, *p < 0.05, **p < 0.01).

As can be seen from Figure 1, compared to the blank group, cell viability treated with RP is almost at the same level, indicating that RP has no cytotoxicity and can be used for treatment.

#### 3.1.2 Experimental analysis of immunofluorescence staining

**FIGURE 2:**
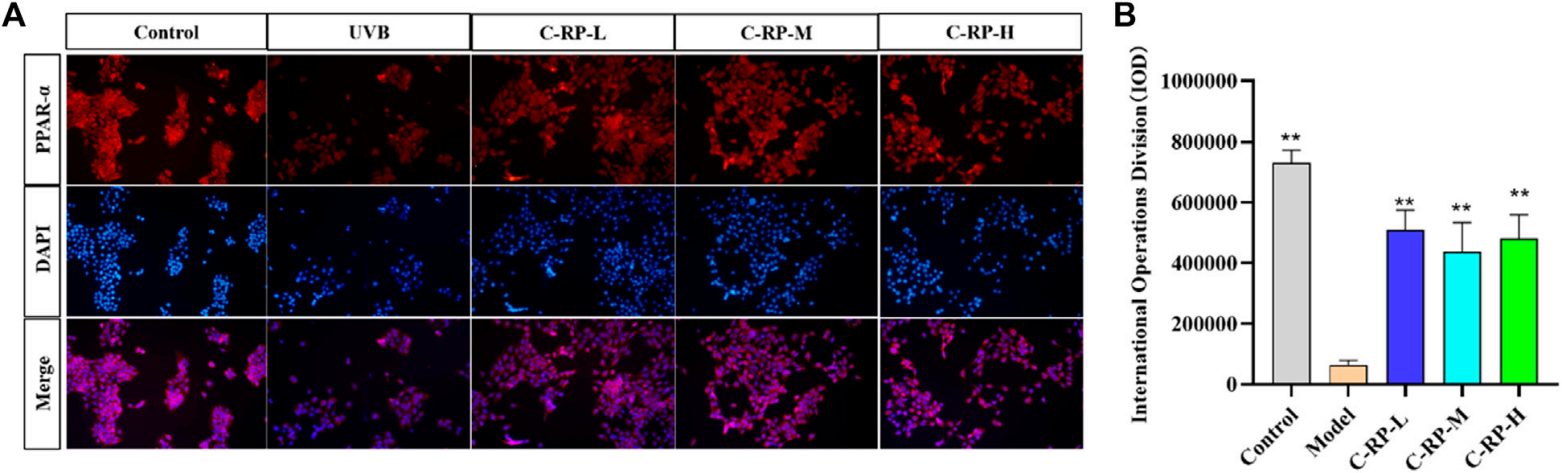
Immunofluorescence analysis. **(A)** Cellular immunofluorescence map. **(B)** IOD value of the cells. (Compared to the model group, *p < 0.05, **p < 0.01).

PPAR-α (peroxisome proliferator-activated receptor-α) is a nuclear receptor and a member of the PPAR family. PPAR-α plays a protective role in skin photoaging. It is able to slow down the process of skin photoaging by inhibiting inflammatory responses, reducing oxidative stress, promoting DNA repair and regulating cellular functions. Immunofluorescence staining was used to analyze the effect of RP on cells. As shown by the immunofluorescence analysis in Figure 2, the expression of PPAR-α and the number of cells were significantly reduced in the UVB model group compared with the blank group. After treatment with different concentrations of RP, the expression of PPAR-α and the number of cells in the C-RP-L, C-RP-M, and C-RP-H groups were significantly increased (*p* < 0.01), and all of them were higher than the model group. It indicated that RP could promote cell proliferation and thus repair the photodamage caused by UVB to the skin by affecting the signaling pathway of PPAR-α.

#### 3.1.3 Experimental analysis of migration cracks

**FIGURE 3:**
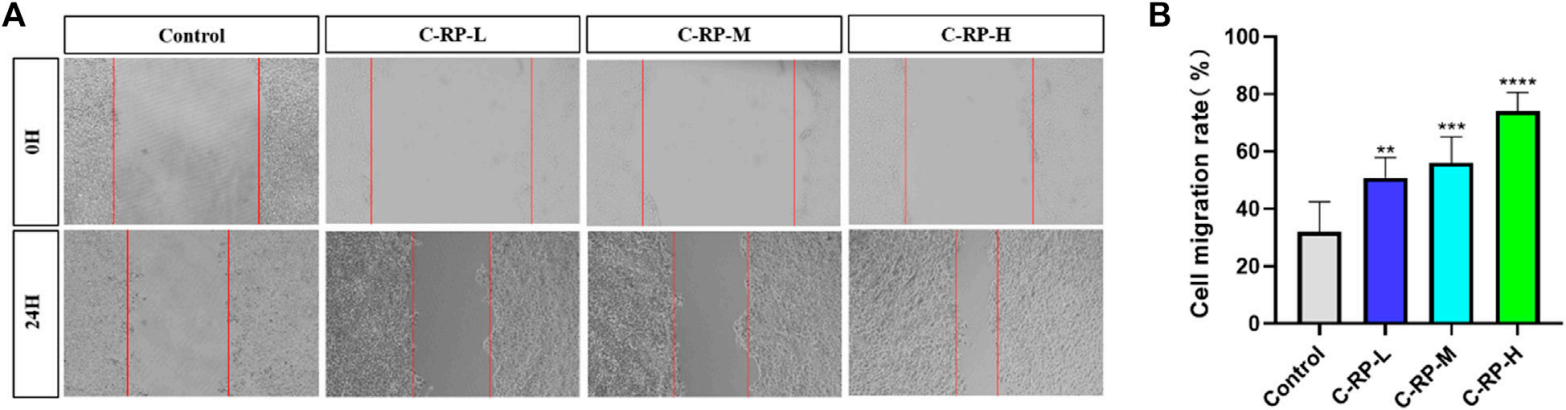
Migrate scratch analysis. **(A)** Cell migration map. **(B)** Cell mobility. (Compared to the control group, *p < 0.05, **p < 0.01)

Cell migration plays an important role in UVB skin photoaging. After the skin has been irradiated by UVB, the skin tissue is damaged and cell migration is a key part of the skin tissue repair and regeneration process. Damaged skin cells are relocated to the damaged area by migratory means and participate in the process of tissue repair. These cells may include stem cells, epidermal cells, and basement membrane cells, among others, which fill in the damaged areas by cell migration to promote tissue repair and regeneration. As shown in Figure 3, after the administration of RP, the cell migration rate of the administered group was significantly higher than that of the blank group, and with the increase of the concentration of RP, the cell migration rate also increased. The results of the cell migration assay indicated that RP could promote the expression of cell growth factors, which in turn promoted the repair of the skin, demonstrating the good tissue repair ability of RP in combating photoaging and restoring skin vitality to play an anti-aging role.

### 3.2 Animal experimental analysis

#### 3.2.1 Skin characterization analysis

**FIGURE 4:**
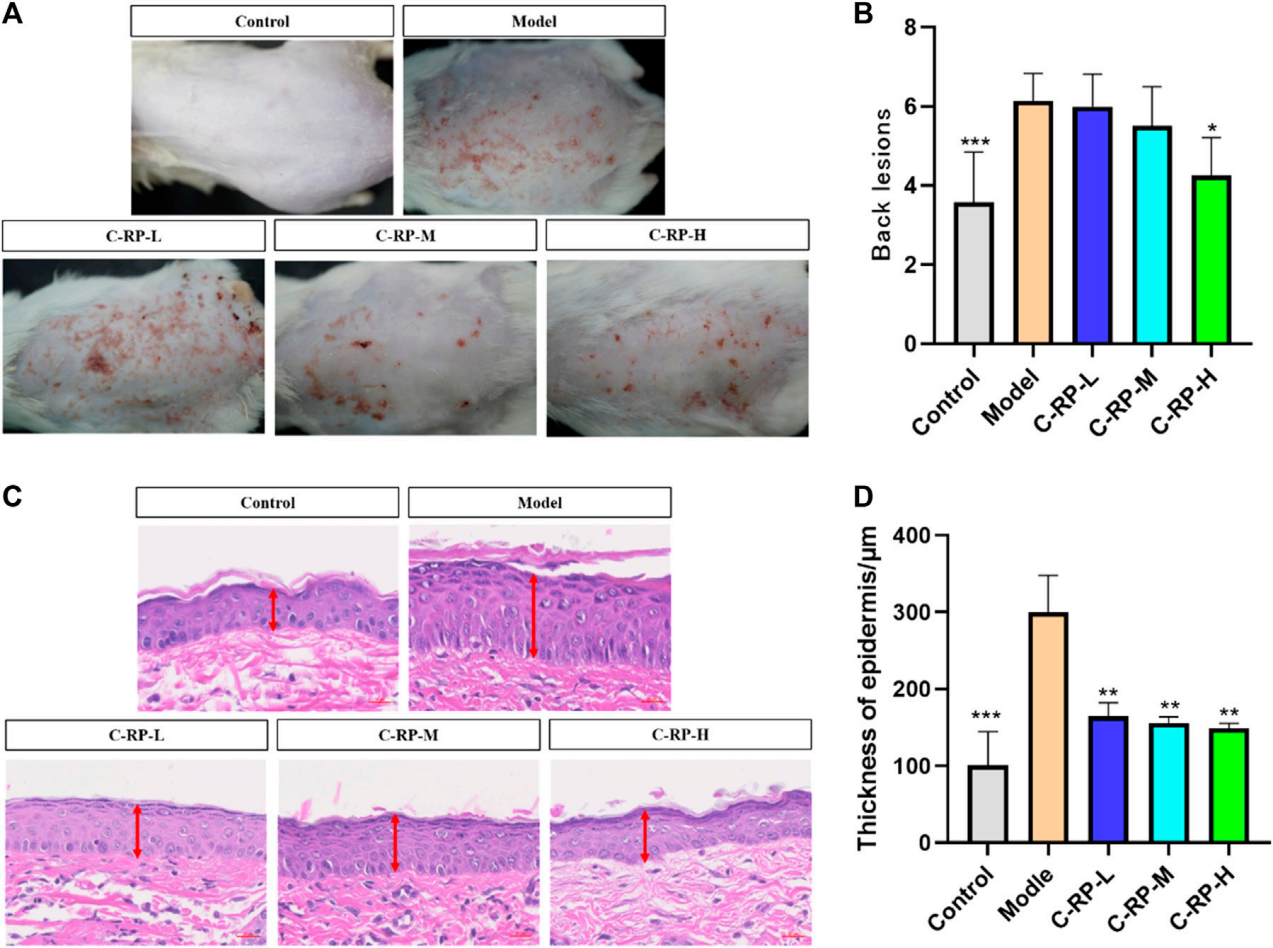
Animal experimental analysis of retinol palmitate. **(A,B)** Appearance skin evaluation, **(C,D)** Skin HE staining test results (Compared with model group, *p < 0.05, **p < 0.01).

Skin surface wrinkles and erythema are often used to assess the severity of UVB-induced photoaging. Figure 4A shows the differences in wrinkles and erythema in the dorsal skin of mice in the blank, model, and administered groups, respectively. As seen in Figure 4A, the wrinkles and erythema in the backs of the mice in the model group after UVB irradiation were significantly more than those in the blank group, whereas those in the backs of the mice in the administered group were significantly less than those in the model group, and the wrinkles and erythema in the backs of the mice in the administered group were reduced with the increase in the concentration of RP administration, which resulted in a significant improvement effect. Figure 4B shows the results of statistical analysis after Graphpad software scoring of wrinkles and erythema appearing on the backs of mice. The photoaging scores on the backs of mice in the model group were significantly higher than those in the blank group, whereas the photoaging scores of mice treated with different RP administration concentrations were significantly lower than those in the model group, and the most significant reduction was observed in the C-RP-H group (*p* < 0.05). Therefore, it can be concluded that RP has a restorative or protective effect on photoaging in mouse skin.

Since the wavelength of UVB was short and its frequency was high, a small dose can have a strong photon energy and cause telangiectasia (Afaq and Mukhtar, 2006), even resulting in erythema (Ulm and Nagy, 2005). Prolonged exposure can induce keratinocyte proliferation, thus leading to skin hyperplasia, roughness, and wrinkles (Yaar and Gilcherst, 2007). Epidermal hyperplasia is one of the characteristics of skin injuries caused by allergic reactions and is commonly used as an indicator to assess the inhibitory effect of drugs on epidermal hyperplasia. As shown in the histological analysis of the tested skin in Figures 4C, D, the allergic reaction after UVB irradiation could lead to a significant increase in epidermal thickness compared with the blank group. Compared with the model group, the C-RP-L, C-RP-M, and C-RP-H groups all reduced the epidermal thickness of mouse skin (*p* < 0.01), indicating that topical application of RP could effectively inhibit the epidermal hyperplasia induced by UVB-induced skin sensitization.

#### 3.2.2 Experimental analysis of toluidine blue staining

**FIGURE 5:**
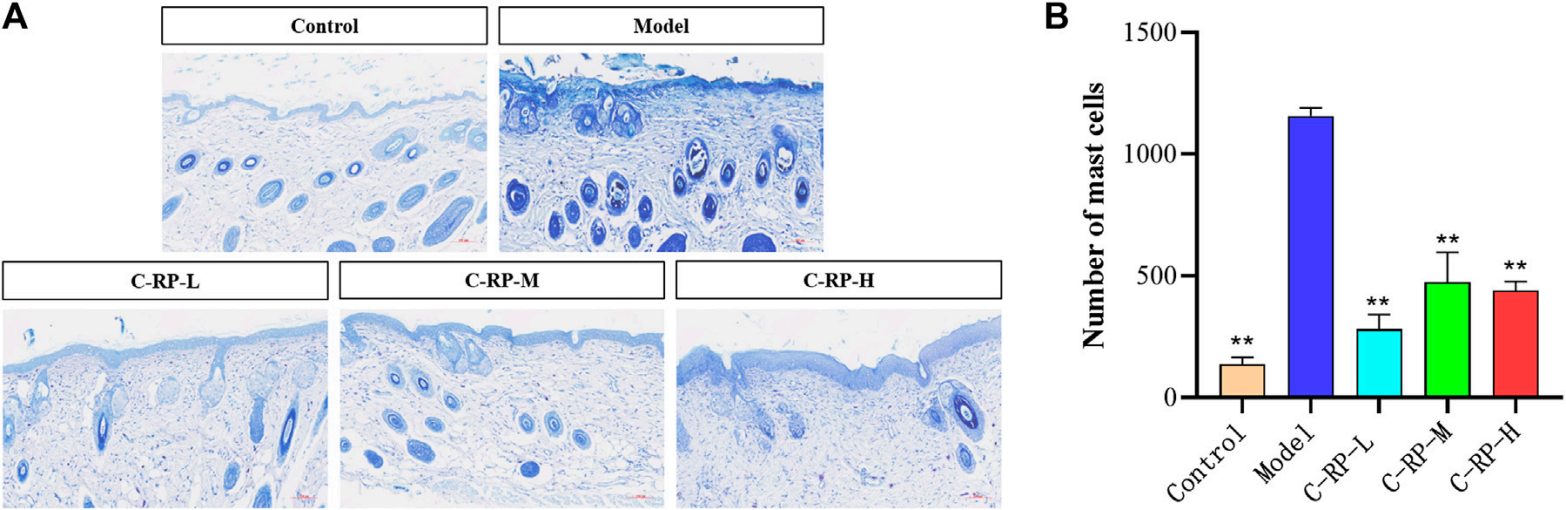
Animal experimental analysis of retinol palmitate. **(A,B)** Skin toluidine blue staining test results (Compared to model group, *p < 0.05, **p < 0.01).

Mast cells are immune cells that are found in a variety of tissues, including the skin. They play an important role in the skin in regulating the immune response and inflammatory processes. UVB irradiation can cause the activation of mast cells and the release of mediators and chemicals stored in their cells. As shown in Figure 5, the blue-purple dots are mast cells in the tissues. The control group untreated with UVB irradiation had a low number of mast cells, while the model group treated with UVB irradiation showed a significant increase in the number of mast cells. Therefore, we believe that UVB induces inflammation in the skin and increases the number of mast cells. As for the RP-treated C-RP-L, C-RP-M, and C-RP-H groups, the number of mast cells in their tissues was significantly reduced compared to the model group (*p* < 0.01). It indicated that RP inhibited the development of UVB-induced skin inflammation and had a strong anti-photo-aging effect.

#### 3.2.3 Masson staining analysis

**FIGURE 6:**
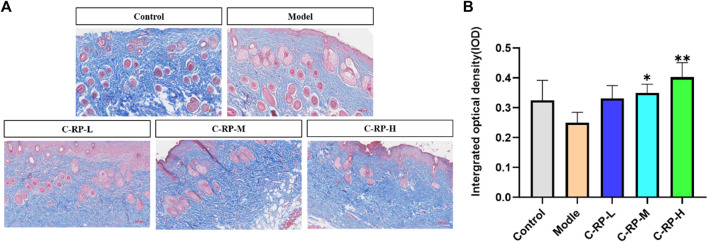
Masson staining results. **(A,B)** Test the results of skin toluidine blue staining (Compared with the model group, *p < 0.05, **p < 0.01).

UVB penetrates directly into the epidermis, which in turn damages collagen fibers in the skin tissue. The collagen fiber content in the skin tissue can be used to evaluate the therapeutic effect of drugs on photodamage caused by UVB. The blue part of Masson stained sections shows collagen fibers, and the red part shows muscle fibers. As shown in Figure 6A, the collagen fibers in the back skin of mice in the blank group were tightly and neatly arranged, whereas in the model group after UVB irradiation, the collagen fibers in the blue portion of the skin tissues were significantly reduced and disordered, and some collagen fibers were also broken. The number of collagen fibers in the back tissues of mice in the C-RP-L, C-RP-M, and C-RP-H groups treated with RP was significantly higher compared with the model group. As can be seen in Figure 6B, the IOP values were significantly increased after the application of RP compared to the model group. Thus, it can be demonstrated that local application of RP can effectively repair the damage of collagen fibers after UVB irradiation.

#### 3.2.4 Immunohistochemical analysis

The cytokines that play an important role in the pathological mechanism of skin aging are IL-6, IL-1β, TNF-α, and Col-Ⅰ, which play an important role in immune response. Studies have shown that during the aging process of many different species of animals, the levels of IL-6, IL-1β, and TNF-α increased, while the level of Col-Ⅰ decreased. The aging increase of IL-6, IL-1β and TNF-α and the decrease of Col-Ⅰ may be the result of adaptive changes to a series of long-term chronic stress such as oxidation and immune emergency. After binding to their receptors, these cytokines can produce a variety of biological activities in the body, such as affecting cell growth, promoting cell differentiation, and so on. Therefore, the levels of IL-6, IL-1β, TNF-α, and Col-Ⅰ can be used as good indicators of individual aging.

**FIGURE 7:**
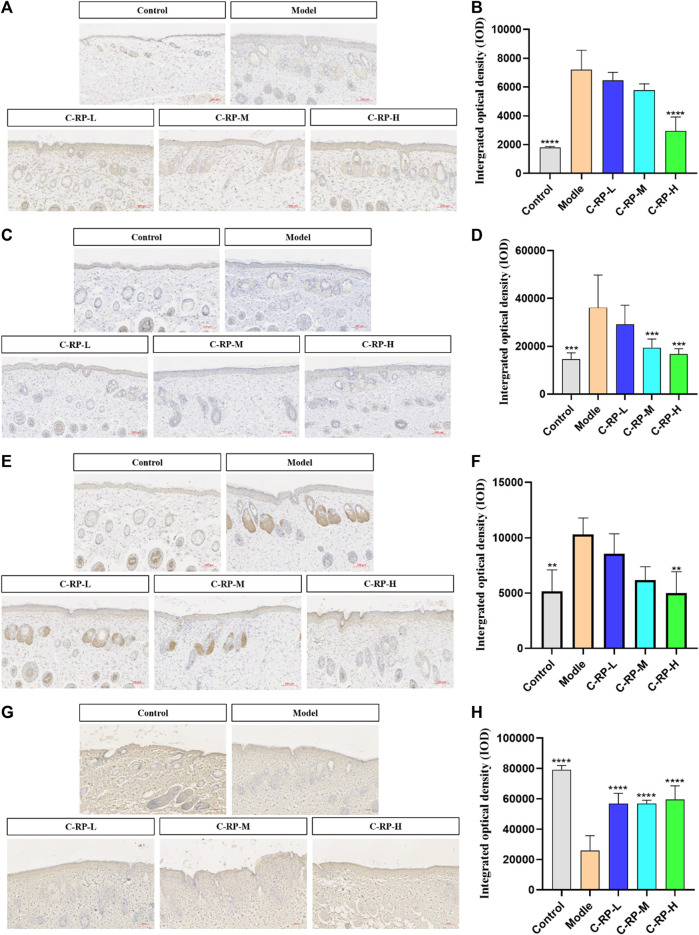
Immunohistochemical test results. **(A,B)** IL-6, **(C,D)** IL-1β, **(E,F)** TNF-α, **(G,H)** Col-I (Compared with the model group, *p < 0.05, **p < 0.01).

As shown in Figure 7, the expression of IL-6, IL-1β and TNF-α was significantly upregulated in all model groups after UVB irradiation compared with the blank group, while the expression of Col-Ⅰ was significantly decreased in the model group compared with the blank group, indicating that the experimental modeling was successful. For all C-RP-L, C-RP-M, and C-RP-H groups treated with different RP concentrations, the expression of IL-6, IL-1β and TNF-α in mouse tissues was significantly decreased in comparison with the model group, while the expression of Col-Ⅰ was significantly increased in comparison with the model group (*p* < 0.01). The results indicated that RP could inhibit the expression of IL-6, IL-1β and TNF-α and promote the expression of Col-Ⅰ to combat UVB photoaging.

### 3.3 Metabolomics, transcriptomics and network pharmacology analysis

#### 3.3.1 Metabonomic analysis

**FIGURE 8:**
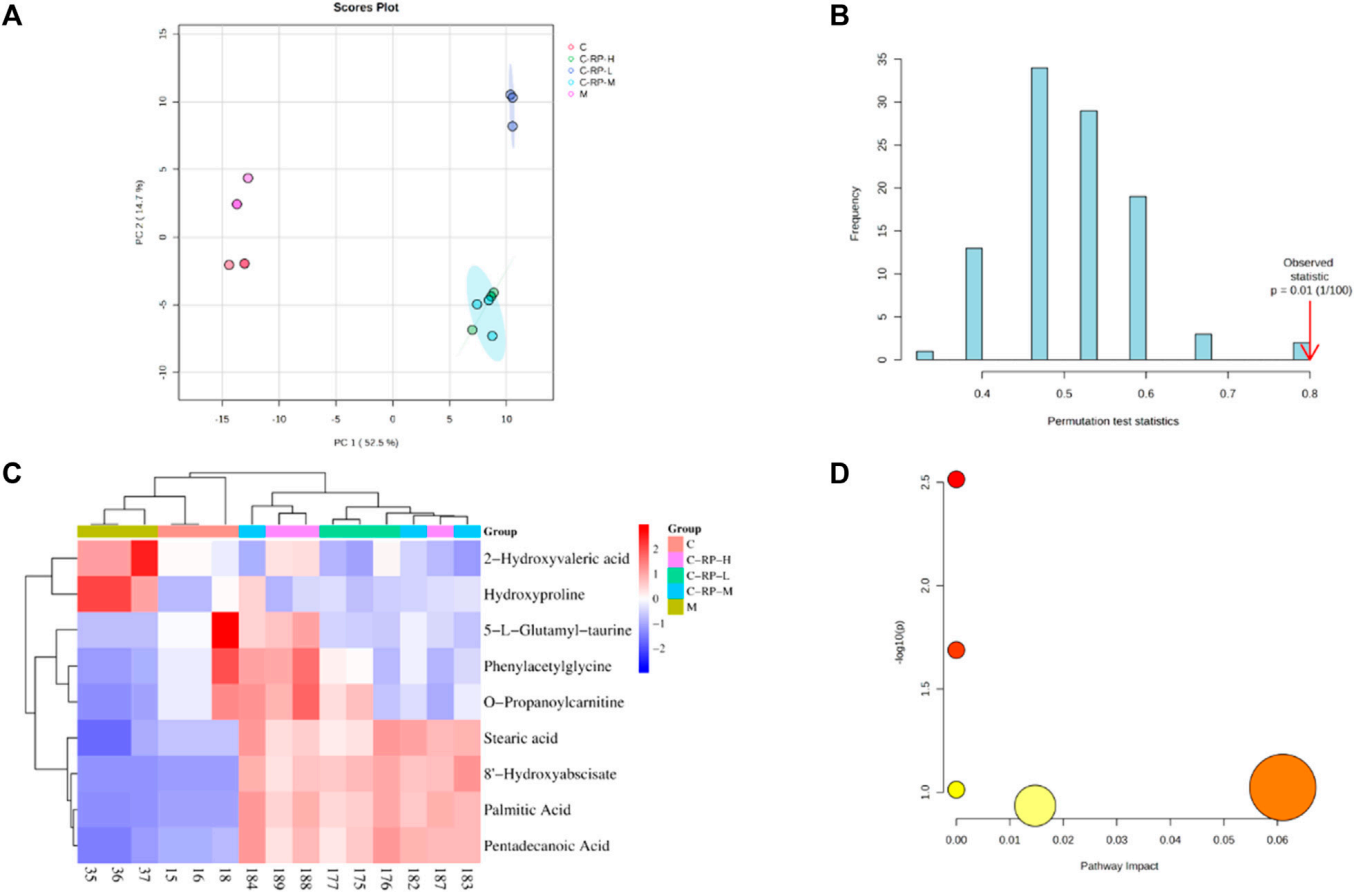
Component analysis of metabolic differentials. **(A)** Principal component analysis and partial least square method were used to distinguish differences in metabolic spectra among different groups. **(B)** The accuracy of the partial least squares model is verified by permutation testing. **(C)** The heat map shows the relative levels of different metabolites after treatment with three different concentrations of RP. **(D)** The bubble diagram shows metabolic pathways rich in different metabolites.

To evaluate the effect of RP on blood metabolism during photoaging in mouse skin, we performed metabolomic analysis of different concentrations. First, principal component analysis (PCA) was performed on all samples, as shown in Figure 8A, the control and RP groups were clearly divided into two clusters, indicating that the modeling was successful. According to Figure 8B, permutation test is used to verify the validity of the PCL model, and the results after 100 permutations prove that the model has good predictive ability. Finally, as shown in Figure 8C, a total of 9 DMSs were identified. Compared to the control group, the expression of 2-Hydroxyvaleric acid and Hydroxyproline was upregulated in the model group, and the expression of 5-L-glutamyl-taurine, phenylacetylglycine, O-propanoylcarnitine, stearic acid, 8- hydroxyabscisate, palmitic acid, and pentadecanoic acid was downregulated. However, most of these metabolites were reversed after treatment by RP, suggesting that these metabolites can regulate metabolic disruption to a certain extent sang. These DMS were then introduced into a metabolic analyzer to analyze metabolic pathways. As shown in Figure 8D, a total of four metabolic pathways were enriched.

#### 3.3.2 Transcriptome analysis

**FIGURE 9:**
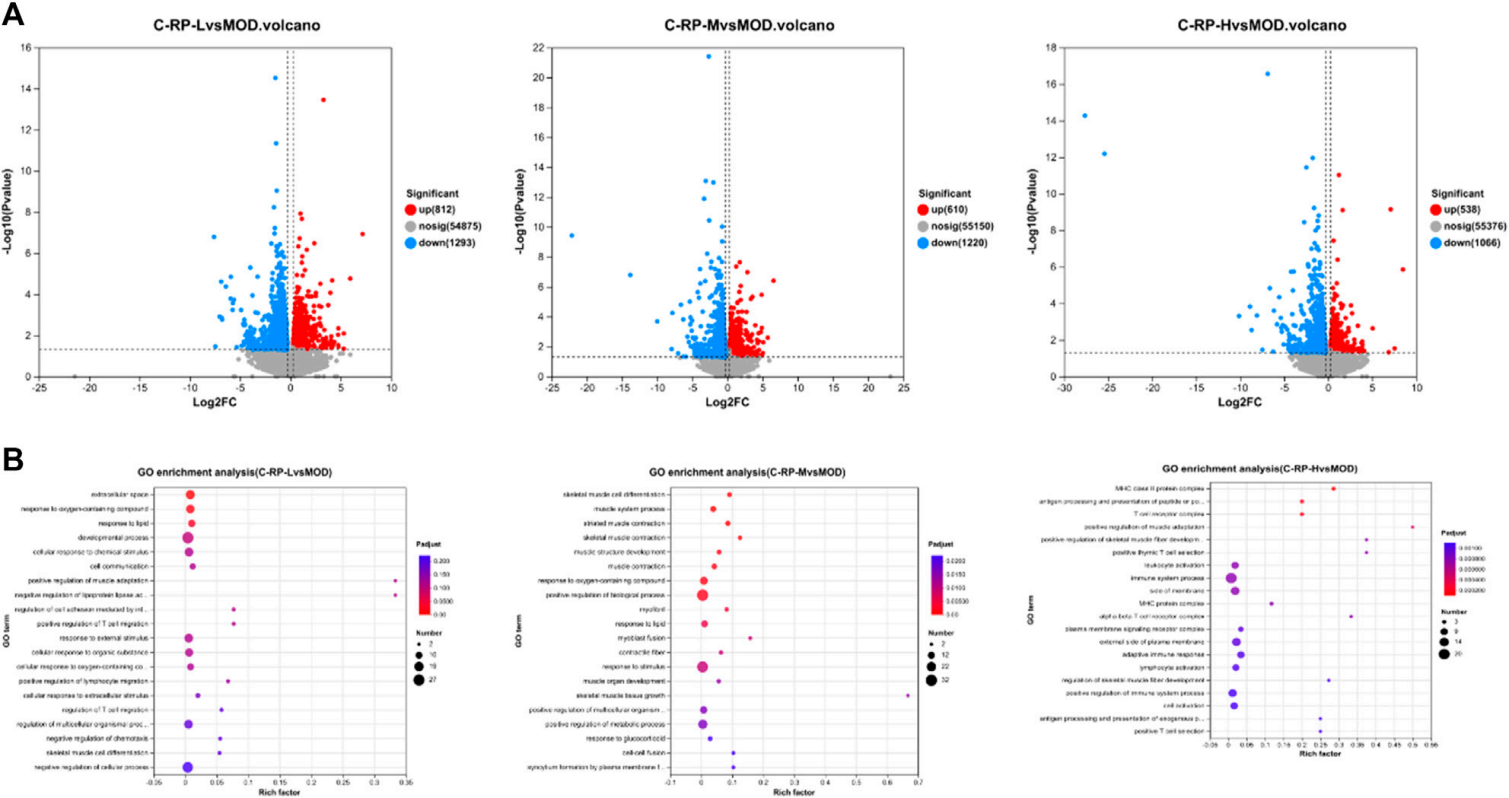
**(A)** The total number of genes detected in the differential grouping and the number of significantly upregulated and downregulated differential genes were shown on the volcano map. **(B)** A total of 26 GO terms are listed in terms of biological processes, cellular components and molecular functions.

Differences in genetic screening criteria defaulted to *p* < 0.05 and |log2 FC| > 1.2, and we compared differentially expressed genes across treatment groups. The volcano map as in Figure 9A shows the total number of genes detected in the differential grouping and the number of significantly upregulated and downregulated differential genes. The horizontal axis of the volcano map shows the change of gene expression ratio, and the vertical axis shows the level of gene significance. Red dots represent upregulated differential genes, blue dots represent downregulated differential genes, and gray dots represent non-differential genes. As can be seen from Figure 9A, the upregulated genes (812) and downregulated genes (1293) in the C-RP-L group were significantly different from those in C-RP-M and C-RP-H.

As shown in Figure 9B, the degree of enrichment of GO genes is represented by a bubble diagram. The dots in the image are positively correlated with the number of pathways for enriching differential genes. The larger the dots, the more ways to enrich differential genes. The redness of the dot is positively correlated with the degree of enrichment, and the redder the dot is, the more obvious the degree of enrichment is.

#### 3.3.3 Network pharmacology analysis

**FIGURE 10:**
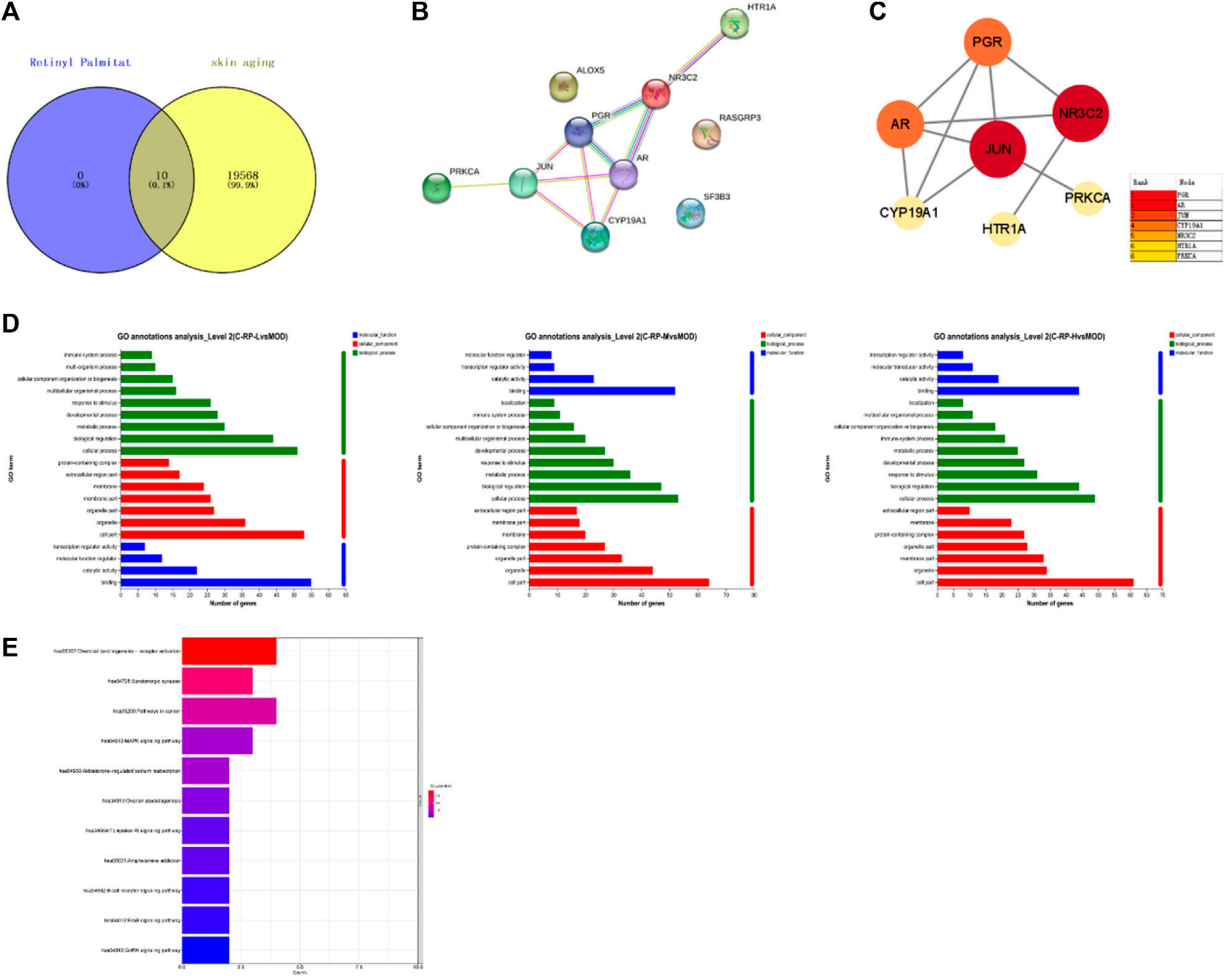
Pharmacological analysis of RP network. **(A)** Venn chart showed the intersection of RP targets and skin aging. **(B,C)** PPI represented interaction between 10 common targets, of which 7 core targets were shown. **(D,E)** Gene ontology function and KEGG enrichment. 20 GO terms in biological processes, cellular composition and molecular function, and the top 11 KEGG pathways of 10 targets were presented.

In order to fully elucidate the potential mechanism of RP against UVB-induced photoaging, an interaction network based on metabolomics and network pharmacology was established. As shown in Figures 10A, B, we identified 10 common targets by crossing 19,578 skin senescence targets and 10 predicted retinol palmitate targets, and then performed PPI analyses on the 10 targets to elucidate the potential interactions between these targets. As shown in Figure 10C, PGR, AR, JUN, CYP19A1, NR3C2, HTR1A and PRKCA may be central targets of flavol palmitate against UVB-induced photoaging. As shown in Figure 10D. There are 10 HUB terms for biological processes, cellular components, and molecular functions. Through biological process analysis, it is found that the key target genes are mainly involved in intracellular steroid receptor signaling pathway, positive regulation of RNA polymerase II promoter on pri-miRNA transcription, positive regulation of endothelial cell proliferation, protein signal transduction, MAPK cascade, *etc.* The results of cell composition analysis suggest that they are related to nucleoplasm, chromatin and perinuclear region. Related molecular function items include steroid binding, RNA polymerase II transcription factor activity, ligand activated sequence, zinc ion binding, g iron ion binding, enzyme binding, *etc.* As shown in Figure 10E, a total of 11 signal pathways were screened by KEGG analysis based on DAVID database. According to the sequence of enrichment genes in-LogP and each pathway, palmitate enrichment pathways of retinol mainly include mitogen-activated protein kinase and ErbB signaling pathway. The target is JUN, PRKCA, RASGRP3. In addition, pathways and targets related to skin photoaging were screened, and RP was found to have 10 common targets involved in the regulation of 11 signal pathways. Seven of these are core targets, and the pathways involved in regulating skin photoaging are mitogen-activated protein kinase and ErbB signaling pathway.

## 4 Conclusion

Retinol palmitate is a derivative of vitamin A, also known as vitamin A palmitate, which is easily absorbed by the skin and then converted into retinol. The main function of retinol is to accelerate skin metabolism, promote cell proliferation and stimulate collagen production. Retinol also has a certain effect on the treatment of acne. Many classic brands and products regard this ingredient as the first choice for anti-oxidation and anti-aging, and it is also an effective anti-aging ingredient recommended by many dermatologists in the United States. The existing whitening products add this ingredient and obtain a patent.

Several early studies have shown that retinoic acid has anti-aging activity on skin; for example, retinoic acid not only has antioxidant activity, but also can induce epidermal thickening, inhibit UV-induced matrix metalloproteinases, and promote collagen synthesis (UV-induced premature aging of skin) (Kong et al., 2016; Schiltz et al., 1986; Dai et al., 2022).

In this study, the role and mechanism of RP on UVB-induced skin photoaging are investigated. Cellular experiments indicate that RP can promote the proliferation of HaCat cells by up-regulating the expression of PPAR-α and facilitate cell migration, thus achieving the efficacy of skin repair. Animal experiments demonstrate that RP can effectively reduce epidermal hyperplasia brought about by UVB irradiation, as well as reduce the number of mast cells in the tissues. RP can also increase the number of collagen fibers in the tissues, effectively repairing the tissue damage brought about by UVB irradiation. IL-6, IL-1β, TNF-α and Col-I are the important cytokines in the pathological mechanism of skin aging. Immunohistochemical analysis suggests that RP can effectively counteract the effects of skin photoaging brought about by UVB irradiation by down-regulating the expression of IL-6, IL-1β and TNF-α and up-regulating the expression of Col-I. Metabolomics, transcriptomics, and network pharmacology analyses show that RP can inhibit skin photoaging process by regulating biological processes such as metabolites and related signaling pathways. Combating skin photoaging is a current research hotspot, therefore, this study reveals the effective therapeutic effects of RP on skin photoaging and its mechanism, which enables the potential application of RP to skin anti-photoaging and therapy.

## Data Availability

The raw data supporting the conclusion of this article will be made available by the authors, without undue reservation.
